# Free-Breathing Liver Magnetic Resonance Imaging With Respiratory Frequency-Modulated Continuous-Wave Radar-Trigger Technique: A Preliminary Study

**DOI:** 10.3389/fonc.2022.918173

**Published:** 2022-06-01

**Authors:** Xinyue Liang, Zhenghong Bi, Chun Yang, Ruofan Sheng, Xinyuan Xia, Zheng Zhang, Yongming Dai, Mengsu Zeng

**Affiliations:** ^1^ Shanghai Institute of Medical Imaging, Fudan University, Shanghai, China; ^2^ Central Research Institute, United Imaging Healthcare, Shanghai, China; ^3^ Department of Radiology, Zhongshan Hospital, Fudan University, Shanghai, China

**Keywords:** magnetic resonance imaging, free-breathing, liver, T2-weighted imaging, diffusion-weighted imaging, respiratory-trigger

## Abstract

**Purpose:**

The aim of this study is to evaluate the performance of free-breathing liver MRI with a novel respiratory frequency-modulated continuous-wave radar-trigger (FT) technique on T2-weighted imaging (T2WI) and diffusion-weighted imaging (DWI) for both healthy volunteers and patients in comparison to navigator-trigger (NT) and belt-trigger (BT) techniques.

**Methods:**

In this prospective study, 17 healthy volunteers and 23 patients with known or suspected liver diseases were enrolled. Six sequences (T2WI and DWI with FT, NT, and BT techniques) were performed in each subject. Quantitative evaluation and qualitative assessment were analyzed by two radiologists. Overall image quality, blurring, motion artifacts, and liver edge delineations were rated on a 4-point Likert scale. The liver and lesion signal-to-noise ratio (SNR), the lesion-to-liver contrast-to-noise ratio (CNR), as well as the apparent diffusion coefficient (ADC) value were quantitatively calculated.

**Results:**

For volunteers, there were no significant differences in the image quality Likert scores and quantitative parameters on T2WI and DWI with three respiratory-trigger techniques. For patients, NT was superior to other techniques for image quality on T2WI; conversely, little difference was found on DWI in qualitative assessment. The mean SNR of the liver on T2WI and DWI with BT, NT, and FT techniques was similar in patients, which is in line with volunteers. FT performed better in terms of higher SNR (705.13 ± 434.80) and higher CNR (504.41 ± 400.69) on DWI at b50 compared with BT (SNR: 651.83 ± 401.16; CNR:429.24 ± 404.11) and NT (SNR: 639.41 ± 407.98; CNR: 420.64 ± 416.61) (*p* < 0.05). The mean ADC values of the liver and lesion with different techniques in both volunteers and patients showed non-significant difference.

**Conclusion:**

For volunteers, the performance of T2WI as well as DWI with three respiratory-trigger techniques was similarly good. As for patients, FT-DWI is superior to BT and NT techniques in terms of higher lesion SNR and CNR at b50.

## Introduction

MRI has become an essential modality for abdominal imaging, as it is sensitive to detect and characterize hepatic lesions with super tissue contrast (1). In the scope of abdominal MRI, T2-weighted imaging (T2WI) and diffusion-weighted imaging (DWI) in combination have been considered as a useful tool to detect and diagnose hepatic lesions and evaluate therapy responses (2).

Motion of the subjects during the MRI examinations often causes severe artifacts in the images. Compared to most types of physiological motion, the temporal resolution of MRI is lower (3). Respiratory motion, one of the primary issues in abdominal imaging, particularly decreases MR image quality. The negative impact would also affect quantitative analysis with signal intensity (SI) and apparent diffusion coefficient (ADC) values (4–7). Moreover, motion artifacts, such as respiratory motion, cardiac pulsation, and bowel peristalsis, decreased examination efficiency in abdominal MRI. In 2017, Schreiber-Zinaman et al. reviewed the frequency of MR examinations with extra repeated sequences for the liver (8). Motion artifacts were the key problems of repeated sequences, especially occurring on fat-suppressed T2-weighted fast spin echo (T2-FSE-FS) sequence (more than 50% of the repeated sequences due to motion in the study) (8). On the other hand, DWI sequence is also sensitive to motion (9). Several studies have demonstrated that DWI (10–12) as well as T2-FS images (13, 14) were affected by cardiac and respiratory motion of abdomen. Due to motion, the most common consequences are severer motion artifacts, lower subjective quality, and diagnostic blurring for both sequences in abdominal MRI.

Numerous studies have attempted to mitigate or correct motion artifacts by a variety of respiratory compensation techniques. For abdominal imaging, breath-hold is a direct method to “freeze” respiration motion. However, image quality, resolution, and coverage would be sacrificed owing to the limited MR acquisition duration. Moreover, consistent position cannot be guaranteed during the scan (3). In addition, patient compliance might be difficult to achieve in children and patients who may be unable to manage their breath-holds (3). Several approaches have been used to overcome the issue of the breath-hold technique on the imaging platforms, such as pressure-based respiratory belt (an external physiologic monitoring pressure-based sensor, typically affixed to the small specific area of the subject’s chest surface) and liver-lung navigator (tracking the position of the diaphragm with a pencil beam to motion correction). However, the respiratory belt technique increased the patient’s preparation time. Compared to the respiratory belt, the navigator is popular in abdominal imaging due to more accurate physiological information and not needing external monitoring devices. Nevertheless, the accuracy depends on the position on the diaphragm and the quality of the pencil beam navigator. Additionally, the respiratory gating efficiency of the navigator was limited for the data only acquired from the fixed acceptance navigator window. Furthermore, the extremely shallow breathing or low liver signal would negatively affect the image quality with the navigator (15).

In order to improve clinical workflow efficiency and patient comfort, contactless respiration monitoring devices have been explored, such as optical cameras (16), acoustic sensors (17), and radars (18, 19). Due to the high spatial accuracy and the ability to detect small movements, frequency-modulated continuous-wave (FMCW) radar has been widely used to track respiratory curves (20). Compared with camera-based respiratory monitoring device, FMCW has notable advantages in terms of illumination conditions, uniform performance across people of all skin types, privacy, and the ability to penetrate objects (21–23). Moreover, FMCW showed high potential to be used as a novel non-contact respiratory trigger in the MR system. The availability of FMCW radar-guided abdominal MRI was reported recently (24). Wang et al. combined the radar with MR scanner for the first time and found that the image quality between FMCW and the respiratory belt on T2WI in volunteers was similar, focusing on the single population group and qualitative analysis (24). With the benefits of improved patient comfort and not needing any extra monitor with subjects, the FMCW-trigger (FT) technique could be potentially used as a respiratory trigger in liver MRI and other medical imaging modalities (24). However, the clinical feasibility of MRI with FT in patients and the performance of FT compared with the navigator-trigger (NT) technique are still unknown.

To our knowledge, this study is the first to evaluate the performance of liver MRI with the FT technique compared to NT and belt-trigger (BT) techniques on T2WI and DWI in both healthy volunteers and patients.

## Materials and Methods

### Study Subjects

This single-center prospective study was approved by the institutional review board. Seventeen healthy volunteers were enrolled in this study between December 2021 and February 2022. None of the volunteers had a known history of liver disease, alcohol abuse, or abdominal surgery. Moreover, twenty-three patients were referred for MRI of the upper abdomen. Patients fulfilled the inclusion criteria: a known or suspected hepatopathy disease and without any contraindication to MRI.

### MR Imaging Protocol

MR examinations were performed on a clinical 1.5-T MR scanner (uMR 680; United Imaging Healthcare, Shanghai, China) with a dedicated 12-channel body array coil and a 32-channel spine array coil. MRI sequences including T2-FSE-FS and DWI with three respiratory-trigger techniques (BT, NT, and FT) in the transverse direction were used with parameters as follows: (1) T2-FSE-FS (TR/TE = 2,501–9,997/90 ms, field of view = 380 × 300 mm, matrix = 320 × 272, slice number = 28, slice thickness/gap = 6/1.8 mm, bandwidth = 260 Hz/pixel, FA = 90°/140°, echo train length (ETL) = 14, acceleration factor = 2) and (2) DWI (TR/TE = 1,950–8,257/66.2 ms, field of view = 380 × 300 mm, matrix = 144 × 144, slice number = 28, slice thickness/gap = 6/1.8 mm, bandwidth = 2200 Hz/pixel, FA = 90°, ETL = 56, acceleration factor = 2, b-value = 50 and 800 s/mm^2^). The imaging parameters were identical for three respiratory-trigger techniques (BT, NT, and FT), apart from TR (varied depending on the subjects’ respiratory cycle). Acquisition parameters of NT were set as follows: navigator length = 15 mm, acceptance ratio = 0.38, acquisition window ratio = 0.5. Therefore, six series of MR images, including two MR sequences and three respiratory-trigger techniques, were acquired for each participant.

**Figure 1:**
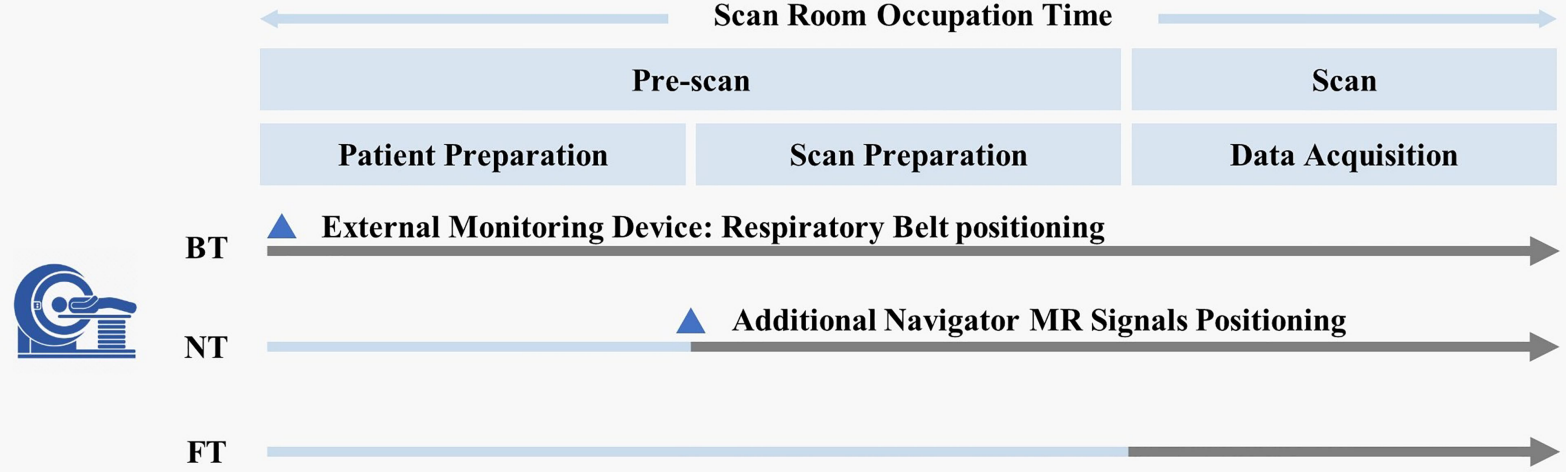
Schematic illustration of the MRI workflow.

In the FT technique, integrated FMCW radar (bandwidth = about 4 GHz) was used to acquire physiological signals from global movement of subjects during MRI. After accurately positioning the abdomen, real-time respiratory curves were reconstructed automatically by automated algorithms (24). Moreover, the clinical workflows among all the three respiratory-trigger techniques were different (**Figure 1**). For BT, technicians need to set up the external monitoring fixation belt at the beginning of subject positioning on the MR scanner table. For NT, the navigator pencil beam should be placed on the subject’s diaphragm in scan preparation. Compared with BT and NT, FT could automatically reconstruct breathing signals in real time during examination and simplify the number of steps without any extra subject preparation.

### Image Analysis

All MR images were independently analyzed by two radiologists (5 and 10 years of experiences in abdominal imaging, respectively) who were blinded to the techniques and clinical information. Qualitative and quantitative analysis were performed for all participants by the same radiologists.

To evaluate the image quality, four criteria—overall image quality, blurring, motion artifacts, and liver edge delineations—were graded on a 4-point Likert scale (1 = unusable; 2 = moderate; 3 = good; 4 = excellent). All T2WI and DWI images with three respiratory-trigger techniques were assessed. It is worthwhile to be mentioned that qualitative image quality scores of DWI were evaluated based on the combination of both b-value images and ADC maps.

Quantitative evaluation was performed by measuring the average SI of the liver and lesion, and the standard deviation (SD) of background noise.

For volunteers, the SI of liver parenchyma was measured with three 50-pixel circular regions of interest (ROIs) set in the right lobe (avoiding vasculature and prominent artifacts) on the T2WI image. These ROIs of the liver were then transferred to corresponding DW images (b50 and b800) as well as ADC maps. Average values of the SI of three ROIs were recorded as SI (liver) of T2WI, SI (liver) of b50, SI (liver) of b800, and the mean ADC value of the liver. The SD of the background noise was measured with a circular roughly 300-pixel ROI located outside the body. Signal-to-noise ratio (SNR) of the liver on T2WI and DWI (b50 and b800) was analyzed by the following formula:


$$\text{SNR}{\;}_{liver}=\frac{\text{SI}\;\left(\text{liver}\right)}{\text{SD}\;\left(\text{background}\right)}$$

For patients, the ROIs in the liver and in the background followed the same procedure as volunteers. In addition, inclusion of lesions needed to be avoided when drawing the ROIs of the liver. The SI and ADC value of the largest lesion of the patient were measured once, with circular ROI covering the lesion as large as possible. The lesion ROIs were drawn on DW images and then were transferred to others.

SNR of liver parenchyma and lesion, as well as lesion-to-liver contrast-to-noise ratio (CNR) of each patient were calculated as follows:


$$\text{SNR}{\;}_{liver}=\frac{\text{SI}\;\left(\text{liver}\right)}{\text{SD}\;\left(\text{background}\right)}$$

$$\text{SNR}{\;}_{lesion}=\frac{\text{SI}\;\left(\text{lesion}\right)}{\text{SD}\;\left(\text{background}\right)}$$

$$\text{CNR}{\;}_{lesion}=\frac{\text{SI}\;\left(\text{lesion}\right)-\text{SI}\;\left(\text{liver}\right)}{\text{SD}\;\left(\text{background}\right)}$$

Furthermore, each qualitative score, acquisition time, quantitative SNR, CNR, and ADC were reported as mean ± standard deviation.

### Statistical Analysis

The presence of changes from image pairs (three respiratory-trigger techniques on T2WI and DWI) in each population was evaluated. All statistical analyses were processed using SPSS (version 26, IBM, NY). The qualitative scores and quantitative parameters (SNR, CNR, and ADC) of paired images were assessed by Wilcoxon signed-rank test. *p* – value ≤ 0.05 indicated a statistically significant difference. The inter-observer agreement was further assessed by calculating the intraclass correlation coefficients (ICCs) (ICC ≤ 0.40, fair; ICC = 0.41–0.60, moderate; ICC = 0.61–0.80, good; ICC = 0.81–1.00, excellent).

## Results

### Study Population

A total of forty subjects were included. Seventeen volunteers (13 men, 4 women; mean age, 34.6 ± 8.2 years; range, 20–49 years; mean age of men, 35.2 ± 8.2 years; range, 20–49 years; mean age of women, 32.5 ± 9.0 years; range, 20–45 years) were recruited internally. Twenty-three patients (mean age, 53.5 ± 12.8 years; range, 34–76 years) were enrolled in this study, including four women (mean age, 46.3 ± 11.3 years; range, 34–58 years) and nineteen men (mean age, 55.1 ± 12.5 years; range, 34–76 years). All recruited patients were included, with the following diseases: liver cancer (*n* = 19) and hemangiomas (*n* = 4).

### Qualitative Image Analysis

**Figure 2:**
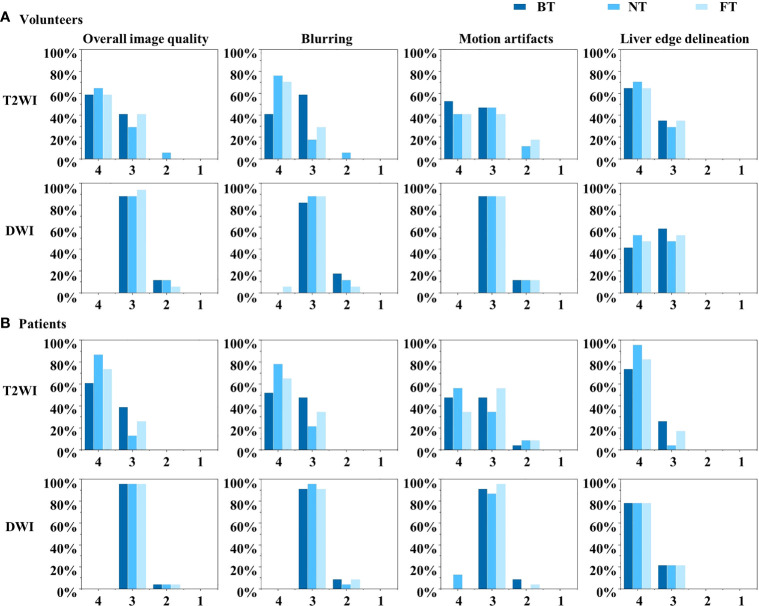
Histograms of the Likert score evaluation of overall image quality, blurring, motion artifacts, and liver edge delineations on T2-weighted imaging (T2WI) and diffusion-weighted imaging (DWI) in **(A)** volunteers and **(B)** patients. The scale ranges from 4 to 1.

**Table 1** and **Figure 2** summarized the qualitative results with three respiratory-trigger techniques (BT, NT, and FT) on T2WI and DWI.

**Table 1:** Pairwise comparisons of qualitative image quality analysis with BT, NT, and FT.

	FT	BT	NT	*p*_(BT vs. FT)_	*p*_(BT vs. NT)_	*p*_(FT vs. NT)_
**Volunteer data (*n* = 17)**						
** T2_FSE_FS**						
** Overall image quality**	3.59 ± 0.49	3.59 ± 0.49	3.59 ± 0.49	>0.999	>0.999	>0.999
** Blurring**	3.76 ± 0.42	3.65 ± 0.48	3.76 ± 0.55	0.414	0.317	>0.999
** Motion artifacts**	3.24 ± 0.73	3.59 ± 0.49	3.41 ± 0.60	0.096	0.102	0.739
** Liver edge delineation**	3.65 ± 0.48	3.65 ± 0.48	3.71 ± 0.46	>0.999	0.317	0.317
** Time (s)**	142.78 ± 27.64	141.26 ± 27.18	179.15 ± 36.10	0.906	0.013	0.007
** DWI**						
** Overall image quality**	2.94 ± 0.24	2.88 ± 0.32	2.88 ± 0.32	0.564	>0.999	0.564
** Blurring**	3.06 ± 0.24	2.88 ± 0.32	2.94 ± 0.24	0.083	0.317	0.157
** Motion artifacts**	2.88 ± 0.32	2.88 ± 0.32	2.88 ± 0.32	>0.999	>0.999	>0.999
** Liver edge delineation**	3.47 ± 0.50	3.41 ± 0.49	3.59 ± 0.49	0.705	0.414	0.655
** Time (s)**	113.09 ± 13.10	111.01 ± 11.49	104.66 ± 9.38	0.532	0.193	0.107
**Patient data (*n* = 23)**						
** T2_FSE_FS**						
** Overall image quality**	3.74 ± 0.44	3.61 ± 0.49	3.87 ± 0.34	0.236	0.013	0.074
** Blurring**	3.65 ± 0.48	3.52 ± 0.50	3.78 ± 0.41	0.236	0.013	0.161
** Motion artifacts**	3.26 ± 0.61	3.43 ± 0.58	3.48 ± 0.65	0.193	0.636	0.014
** Liver edge delineation**	3.83 ± 0.38	3.73 ± 0.44	3.96 ± 0.20	0.492	0.035	0.131
** Time (s)**	129.08 ± 21.86	130.58 ± 57.49	163.26 ± 26.94	0.830	<0.001	<0.001
** DWI**						
** Overall image quality**	2.96 ± 0.20	2.96 ± 0.20	2.96 ± 0.20	>0.999	>0.999	>0.999
** Blurring**	2.91 ± 0.28	2.91 ± 0.28	2.96 ± 0.20	>0.999	0.564	0.317
** Motion artifacts**	2.96 ± 0.20	2.91 ± 0.28	3.13 ± 0.34	0.317	0.025	0.046
** Liver edge delineation**	3.22 ± 0.41	3.22 ± 0.41	3.22 ± 0.41	>0.999	>0.999	>0.999
** Time (s)**	98.63 ± 11.64	97.81 ± 10.94	94.52 ± 14.11	0.390	0.438	0.140

Data are given as mean ± standard deviation. T2-FSE-FS, T2-weighted fast spin echo with fat saturation; DWI, diffusion-weighted imaging; BT, conventional pressure-based respiratory belt-trigger technique, NT, navigator-trigger technique; FT, respiratory frequency-modulated continuous-wave radar-trigger technique.

**Figure 3:**
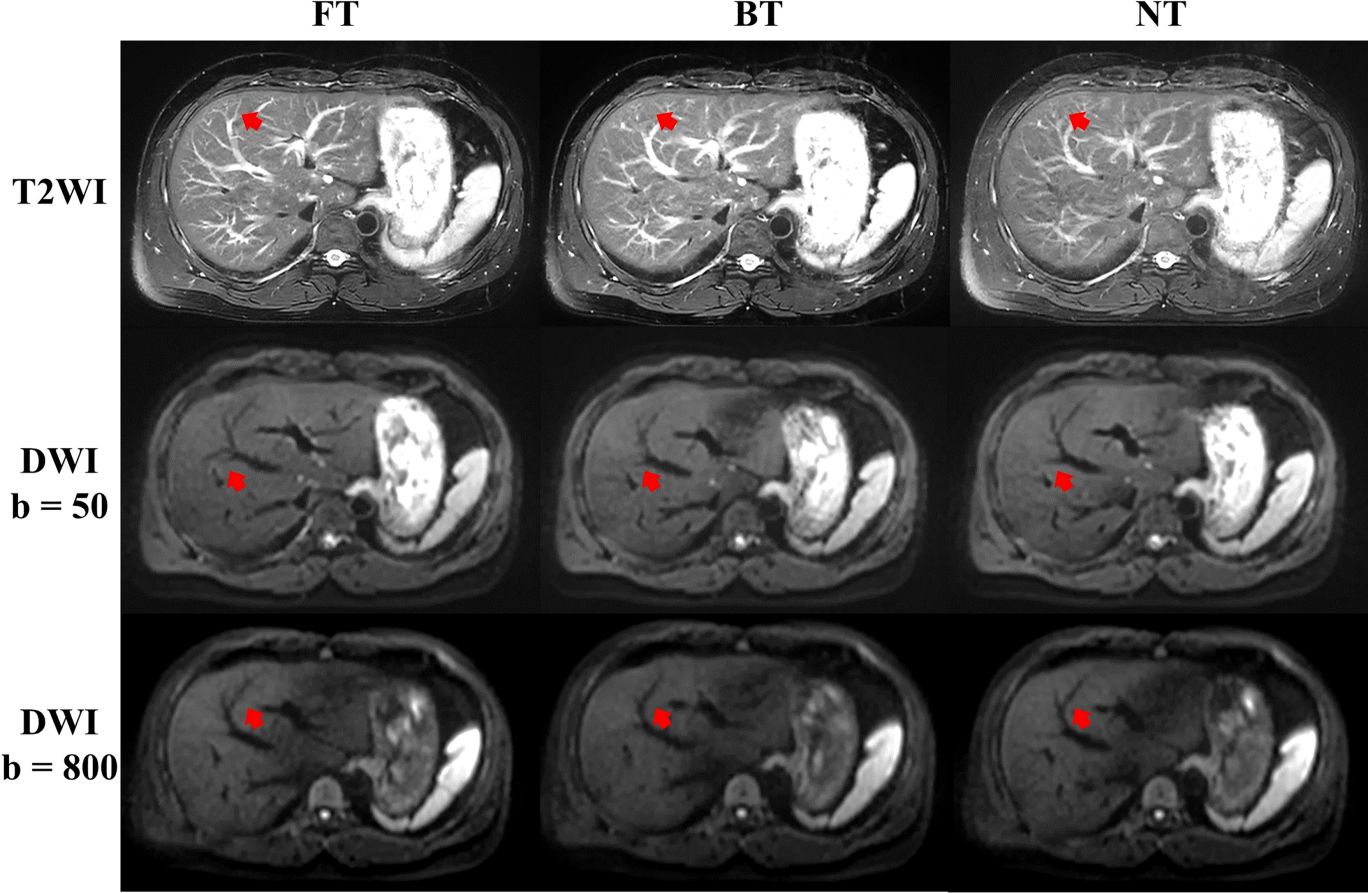
Example abdominal T2-weighted imaging (T2WI) and diffusion-weighted imaging (DWI) of a male volunteer from respiratory frequency-modulated continuous-wave radar trigger technique (FT), respiratory belt-trigger technique (BT), and navigator-trigger technique (NT). The arrow points at vessels indicated better blood vessel visualization with FT on T2WI and DWI.

For volunteers, the paired image quality of abdomen with different techniques was comparable on both T2WI and DWI. As shown in **Figure 3**, FT presented with better blood vessel visualization compared to other techniques on T2WI as well as DWI. Nonetheless, differences were nonsignificant (*p* > 0.05) regarding the qualitative Likert score evaluation in **Table 1**. Average acquisition time of T2WI with NT was significantly longer than BT (*p* = 0.013) and FT (*p* = 0.007). However, no significant differences in average acquisition time were found on DWI sequences (*p* > 0.05).

**Figure 4:**
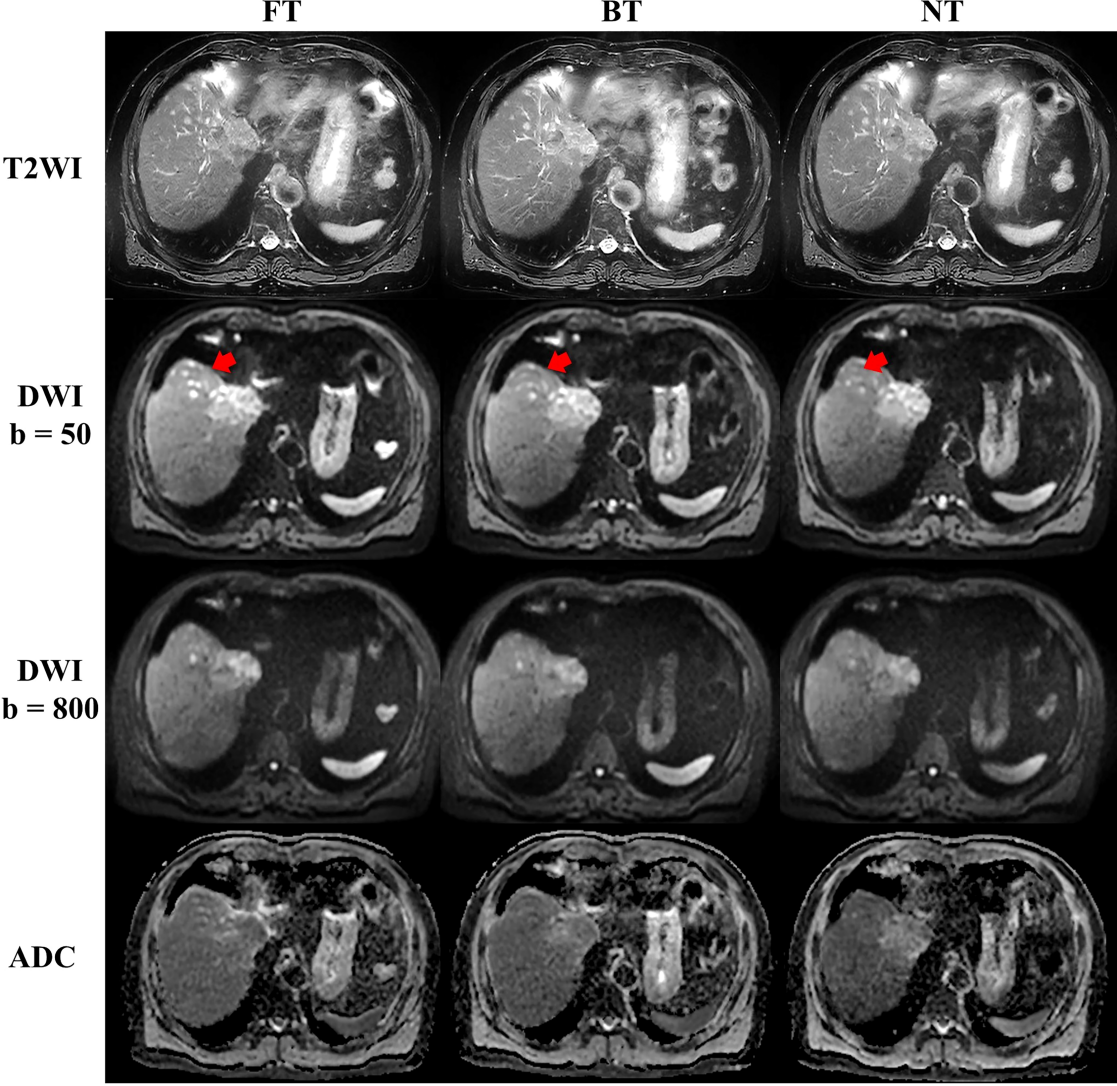
Example abdominal T2-weighted imaging (T2WI) and diffusion-weighted imaging (DWI) of a male liver cancer patient from respiratory frequency-modulated continuous-wave radar trigger technique (FT), respiratory belt-trigger technique (BT), and navigator-trigger technique (NT). The lesion marked by arrow has much higher signal intensity in the FT images.

For patients, NT performed mostly better for image quality on T2WI, followed by FT and BT. In detail, good and excellent overall image quality (Likert score 3 and 4 in **Figure 2**) were seen more often in NT (13.04% and 86.96%) and FT (26.09% and 73.91%) compared to BT (39.13% and 60.87%) on T2WI. Blurring, motion artifacts, and liver edge delineation were superior in NT on T2WI than other techniques, with mirror and no significant differences. An example of a liver cancer patient with better lesion conspicuity at b50 DWI is shown in **Figure 4**. Consistent with the findings of volunteers, mean acquisition time of T2WI with NT was also significantly longer than others (*p* < 0.001) in patients. On DWI, image quality with three respiratory-trigger techniques was not significantly different; however, motion artifact scores were superior in NT compared to BT (*p* = 0.025) and FT (*p* = 0.046). Moreover, the average acquisition time of DWI presented nonsignificant differences (*p* > 0.05).

### Quantitative Image Analysis

Quantitative statistics for the SNR of the liver and lesion are summarized in **Table 2**. For volunteers, the differences in mean SNRs of the liver in paired images on both sequences with three techniques were less than 7%. Mean SNR of the liver was non-significant or slightly higher with BT on T2WI. As for DWI, the mean liver SNR of NT at b50 was significantly higher (*p* < 0.05) in comparison to the SNR of BT and FT. However, no significant differences were seen in the SNR of the liver at b800 in volunteers.

**Table 2:** Pairwise comparisons of SNRs among BT, NT, and FT.

	FT	BT	NT	*p*_(BT vs. FT)_	*p*_(BT vs. NT)_	*p*_(FT vs. NT)_
**Volunteer data (*n* = 17)**						
** T2_FSE_FS**	236.19 ± 71.28	246.40 ± 73.81	242.40 ± 70.11	0.163	0.022	0.463
** DWI**						
** b50**	180.32 ± 84.57	183.32 ± 87.19	191.96 ± 51.77	0.006	0.227	0.019
** b800**	111.61 ± 43.18	113.59 ± 49.16	118.27 ± 50.09	0.084	0.687	0.586
**Patient data (*n* = 23)**						
** T2_FSE_FS**						
** Liver**	178.95 ± 51.60	184.42 ± 57.49	184.68 ± 59.82	0.236	0.738	0.503
** Lesions**	514.97 ± 212.20	499.62 ± 240.53	528.59 ± 256.16	0.059	0.020	0.918
** DWI**						
** b50**						
** Liver**	236.21 ± 112.47	225.47 ± 117.14	223.39 ± 122.60	0.153	0.831	0.033
** Lesions**	705.13 ± 434.80	651.83 ± 401.16	639.41 ± 407.98	0.027	0.600	0.002
** b800**						
** Liver**	127.47 ± 53.47	129.28 ± 51.56	126.61 ± 57.56	0.523	0.523	0.831
** Lesions**	278.36 ± 212.76	283.21 ± 230.67	260.76 ± 203.99	0.539	0.362	0.495

For patients, mean liver SNRs on T2WI showed no statistical differences among three respiratory-trigger techniques (*p* > 0.05). For lesions on T2-FSE-FS images, FT and NT techniques performed similarly good for SNR measurement (*p* > 0.05). Moreover, NT-T2WI resulted in higher SNR value of lesions than BT (*p* = 0.02). On DWI, the mean SNR of the liver in FT was slightly but non-significantly lower compared to BT and NT at b50 trace. However, the mean SNR of liver lesions with FT (705.13 ± 434.80) at b50 images was higher than BT (651.83 ± 401.16, *p* = 0.03) and NT (639.41 ±407.9, *p* = 0.002). At b800 trace, the SNRs of liver parenchyma and lesions were almost equal among paired images.

Similar results were seen for CNR calculations (**Table 3**). Mean CNR of FT and NT on T2WI was comparable (*p* > 0.05). Focusing on DW images at b50, CNR with FT was significantly higher compared to BT (*p* = 0.017) and NT (*p* = 0.021). However, mean CNR presented mirror differences among three techniques at b800, but not statistically significant.

**Table 3:** Pairwise comparisons of CNRs among BT, NT, and FT.

		FT	BT	NT	*p*_(BT vs. FT)_	*p*_(BT vs. NT)_	*p*_(FT vs. NT)_
**T2_FSE_FS**	338.95 ± 194.89		318.24 ± 211.04	347.31 ± 223.65	0.023	0.009	0.891
**DWI**							
** b50**	504.41 ± 400.69		429.24 ± 404.11	420.64 ± 416.61	0.017	0.495	0.021
** b800**	169.53 ± 193.82		176.28 ± 209.58	163.38 ± 183.58	0.187	0.246	0.187

**Figure 5:**
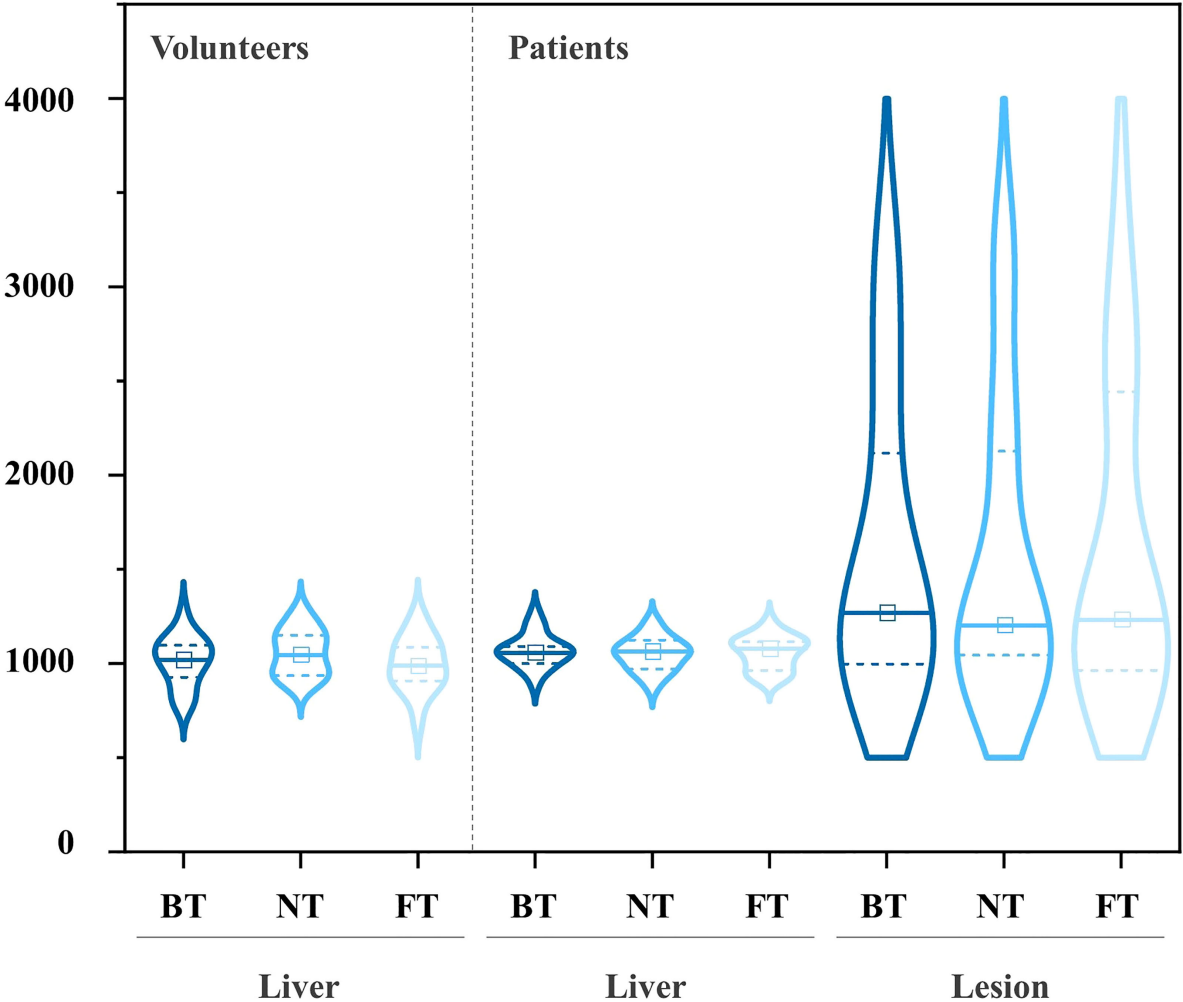
Pairwise comparisons of ADC on DWI in volunteers (left) and patients (right) with the violin plots (central mark indicates the median; two dot lines indicate the 25th percentile and the 75th percentile, from bottom to top).

Mean ADCs of the liver and lesion with different techniques in both populations are shown in **Table 4**. NT showed higher ADCs of the liver in volunteers, but without statistical differences. For patients, significant differences in mean ADC levels were found between lesions and liver parenchyma. Additionally, no significant differences in ADCs among FT, BT, and NT were found in the liver as well as in lesions. Box plots of the ADC values of volunteers and patients are shown in **Figure 5**. The distribution of ADC values from paired images with three respiratory trigger techniques in each group was similar. ADC range of lesions and healthy liver parenchyma overlapped in patients.

**Table 4:** The ADC measurements (μm^2^/s) of DWI among BT, NT, and FT.

	FT	BT	NT	*p*_(BT vs. FT)_	*p*_(BT vs. NT)_	*p*_(FT vs. NT)_
**Volunteer data (*n* = 17)**						
** Liver**	996.61 ± 92.83	1,013.36 ± 128.76	1,058.12 ± 118.06	0.868	0.136	0.093
**Patient data (*n* = 23)**						
** Liver**	1,056.34 ± 86.68	1,069.11 ± 87.23	1,051.31 ± 86.42	0.738	0.648	0.831
** Lesion**	1,606.94 ± 856.33	1,602.11 ± 797.95	1,566.83 ± 782.93	0.838	0.733	0.802

There were almost perfect interobserver agreements between two readers for the image quality analysis (ICC > 0.90).

## Discussion

Numerous motion reduction techniques have been developed in recent years. Nonetheless, not a single technique is available for all kinds of imaging scenarios (3). Patient comfort and optimized workflow are key factors for improving the successful scan rates and MR examination efficiency. BT and NT were the most common motion reduction techniques, while BT needs external monitoring devices (fixation belt) with additional patient preparation time. For NT, the placement of the navigator pencil beam on the subject’s diaphragm would also increase extra preparation time. Additionally, efficiency of NT was limited by the acceptance window. Therefore, it is of great value to investigate the performance of the routine sequences with novel contactless technique especially in liver lesion detection and MR workflow simplification. In this preliminary study, qualitative and quantitative comparisons on DWI and T2WI with different respiratory-trigger techniques in two populations were evaluated respectively.

From qualitative analysis, NT showed better image quality on T2WI in patients. In line with our results, Kim et al. reported that the image quality of T2-FSE with NT was superior to BT in patients, but without significant differences (25). Lee et al. also found that the image sharpness of NT was significantly better than BT in patients (26). These results could partly be explained by the fact that the respiratory signal of the belt (obtained from only part of chest surface movements) was not as accurate as that from the navigator (diaphragmatic movements). Additionally, the image quality on FT-T2WI was improved in comparison with BT-T2WI in patients, although the difference was nonsignificant. As mentioned in the *Introduction* and *Materials and Methods*, FT enables a large field of view for motion determination compared to BT, which may be beneficial to the accuracy and stability of respiratory signal measurement. Similar findings were illustrated in a camera-based respiratory technique compared to BT in cholangiopancreatography MRI (16, 27). It is well known that abnormal respiration occurred more often in patients with liver disease than the healthy volunteers. Moreover, the respiratory belt technique would fail when breathing position drifted and breathing patterns changed (3). In this study, average quality scores were broadly comparable among FT, BT, and NT on T2WI as well as DWI in volunteers and those on DWI in patients. Wang et al. examined healthy volunteers and directly compared FT-T2-FSE with BT-T2-FSE abdominal imaging and found that the image quality scores were equivalent (24), which is in keeping with our findings.

In the quantitative calculation of patients, non-significant differences among the SNR of the liver with FT, BT, and NT techniques were found on both T2WI and DWI sequences. In addition, NT-T2WI performed better than BT and FT in terms of mean SNR of lesion, with non-significant differences. Previous studies have reported conflicting results in patients. Kim et al. showed that healthy liver parenchyma of patients and liver lesion SNRs acquired with BT were significantly higher compared to those with NT on T2WI (28). In contrast, Lee et al. found that higher liver SNR was measured on NT-T2WI than on BT-T2WI, while the performance of lesion-to-liver CNR was not significantly different (26). This difference might be explained by the different MR protocols between NT and BT techniques. As we all know, higher number of acquisitions will increase the SNR, while longer ETL is associated with lower SNR and may lower lesion-to-liver CNR. BT has twice the number of signal averages compared with NT in Kim’s work (28). However, Lee et al. set identical signal averages between BT and NT but the ETL was 44% increased with BT (26).

DWI has been widely used in the detection and characterization of liver lesions. Low b-value DWI is important for the detection of hepatic lesions with the advantage of liver vessel suppression, higher SNR, and being less affected by artifacts including eddy currents or blurring (29). Several studies have shown that low b-value was superior to DWI with higher b-values and T2WI for the detection of liver lesions (30–32). In this preliminary study, the performance was similar between NT and BT in terms of lesion SNR and CNR at b50. Previous studies have also compared the difference in liver lesion detection between BT-DWI and NT-DWI in patients. Takayama et al. (33) reported that CNR between BT-DWI and NT-DWI was non-significantly different with identical MR parameter settings. Bouchaibi et al. demonstrated that overall sensitivity between two DWI sequences was equivalent for the detection of lesions (34). Nevertheless, the performance was only evaluated from the subjective qualitative standards, without quantitative analysis. Choi et al. reported equal performance between NT-DWI and free-breathing DWI without any gating in terms of lesion SNR, CNR at b50, and the sensitivity to detect liver lesions (6). In this study, lesion-to-liver CNR with FT was significantly higher than other techniques, at b50 images. This finding may be a result of the highest SNR of lesion measured at b50 with FT. Additionally, higher lesion SNR and higher CNR can positively increase the conspicuity of liver lesions. The diagnostic performance of lesion characteristics among three techniques may need to be studied further.

It was reported that the mean ADC value might depend greatly on the chosen respiratory technique strategy, like breath-hold and free-breathing (4). However, the ADC obtained in free-breathing with various respiratory-trigger techniques was not significantly different (6, 7). This is in line with our findings that ADC values were nearly identical for BT, NT, and FT techniques on paired images in both volunteers and patients. It was noticed that the ADC value of the right lobe (NT DWI, in volunteers: 1,058.12 ± 118.06 μm^2^/s) seemed to be lower compared with the previous literature, such as 1,387–1,400 μm^2^/s (NT DWI, in volunteers) (7), and it may be explained by ADCs changed with the selection of b-values (35).

Recently, several studies evaluated the time of MRI processes and assessed the association with clinic MR efficiency. For instance, Rooyen et al. quantified that the pre-scan time during the whole MR workflow was almost 8% (36). Streit et al. suggested that positioning of the patient and coils should be more time efficient (37). Abdominal MRI acquisition time was long, even up to 58 min reported at one center (38). Moreover, motion artifacts seriously affect the MRI efficiency and add extra costs to hospitals. Andre et al. estimated that the potential revenue loss of hospital was approximately $115,000 per scanner per year (39). Thus, it is of great value to improve MR exam efficiency in liver imaging. In this study, FMCW radar was used as a respiratory motion trigger integrated into the MR scanner. In comparison with traditional respiratory-trigger techniques, FT could improve MR exam efficiency without additional workload in pre-scan time. Additionally, the respiratory belt technique must be tight enough to capture the patient’s breathing. As a result, conventional contact respiratory belt may cause discomfort, which may increase the likelihood of patient movement. Furthermore, additional inspection time to the radiologist would be increased if motion-corrupted images are not detected or repeated during the MR exams. Moreover, the situation would be worse if essential images are nondiagnostic. It is well known that the primary method to improve MRI efficiency is patient motion reduction. Thus, it is essential for the technician to identify early the incoherent respiration. Moreover, clear feedback on whether the patient is following respiratory instructions or not during acquisitions could easily be obtained from the respiratory curve with FT. Likewise, the liver lesion detection efficiency of radiologists may be improved due to the highest lesion SNR and CNR with FT than other respiratory-trigger techniques at DWI b50 trace. FT may be a potential contactless respiratory-trigger technique in the routine MR examinations with the advantage of improvement of patient comfort and workflow optimization, particularly for children, pregnant women, and the elderly.

This study has several limitations. Firstly, the results of this prospective study were limited by the small sample size, by being a single-center study, and by using a single MR scanner, which may have caused some statistical bias. Secondly, the research group of healthy volunteers (young adults) and patients (middle-aged and older people) may not be perfectly matched. Notably, the quantitative measurements including SNR, CNR, and ADC in young and old populations may differ. Moreover, this study was performed on a 1.5-T system and did not generalize to other higher field strengths, like 3.0 T, at which most studies were currently performed (40). It is worth mentioning that the performance of different respiratory-trigger techniques may differ from different sizes (6, 33, 41) and types (6) of evaluated liver lesions. Additionally, limited kinds of liver disease may influence the generality of the results in this study.

## Conclusion

In conclusion, the performance of T2WI as well as DWI with three respiratory-trigger techniques was similarly good in volunteers. As for patients, FT-DWI is better suited than BT and NT techniques in terms of higher lesion SNR and CNR at b50, without degrading the qualitative and quantitative performance at b800 DWI and T2WI.

## Data Availability Statement

The original contributions presented in the study are included in the article/supplementary material. Further inquiries can be directed to the corresponding author.

## Ethics Statement

The studies involving human participants were reviewed and approved by Zhongshan Hospital of Fudan University. The patients/participants provided their written informed consent to participate in this study. Written informed consent was obtained from the individual(s) for the publication of any potentially identifiable images or data included in this article.

## Author Contributions

XL and YD contributed to the conception and design of the study. ZB contributed to the enrollment of the patients and performed the MRI examination. CY and RS contributed to data analysis. XL, XX, and ZZ contributed to the discussion. XL wrote the manuscript. MZ revised the language and reviewed the manuscript. All authors contributed to the article and approved the submitted version.

## Conflict of Interest

Authors XL, XX, ZZ, and YD were employed by Shanghai United Imaging Healthcare Co., Ltd.

The remaining authors declare that the research was conducted in the absence of any commercial or financial relationships that could be construed as a potential conflict of interest.

## Publisher’s Note

All claims expressed in this article are solely those of the authors and do not necessarily represent those of their affiliated organizations, or those of the publisher, the editors and the reviewers. Any product that may be evaluated in this article, or claim that may be made by its manufacturer, is not guaranteed or endorsed by the publisher.
